# Modulation of Replicative Lifespan in *Cryptococcus neoformans*: Implications for Virulence

**DOI:** 10.3389/fmicb.2017.00098

**Published:** 2017-01-30

**Authors:** Tejas Bouklas, Neena Jain, Bettina C. Fries

**Affiliations:** ^1^Department of Medicine (Division of Infectious Diseases), Stony Brook University, Stony BrookNY, USA; ^2^Department of Biomedical Sciences, Long Island University-Post, BrookvilleNY, USA; ^3^Department of Medicine (Division of Infectious Diseases), Albert Einstein College of Medicine of Yeshiva University, BronxNY, USA; ^4^Department of Molecular Genetics and Microbiology, Stony Brook University, Stony BrookNY, USA; ^5^Department of Microbiology and Immunology, Albert Einstein College of Medicine of Yeshiva University, BronxNY, USA

**Keywords:** fungus, pathogen, aging, virulence, persistence, sirtuin

## Abstract

The fungal pathogen, *Cryptococcus neoformans*, has been shown to undergo replicative aging. Old cells are characterized by advanced generational age and phenotypic changes that appear to mediate enhanced resistance to host and antifungal-based killing. As a consequence of this age-associated resilience, old cells accumulate during chronic infection. Based on these findings, we hypothesized that shifting the generational age of a pathogenic yeast population would alter its vulnerability to the host and affect its virulence. *SIR2* is a well-conserved histone deacetylase, and a pivotal target for the development of anti-aging drugs. We tested its effect on *C. neoformans*’ replicative lifespan (RLS). First, a mutant *C. neoformans* strain (*sir2Δ*) was generated, and confirmed a predicted shortened RLS in *sir2Δ* cells consistent with its known role in aging. Next, RLS analysis showed that treatment of *C. neoformans* with Sir2p-agonists resulted in a significantly prolonged RLS, whereas treatment with a Sir2p-antagonist shortened RLS. RLS modulating effects were dependent on *SIR2* and not observed in *sir2Δ* cells. Because *SIR2* loss resulted in a slightly impaired fitness, effects of genetic RLS modulation on virulence could not be compared with wild type cells. Instead we chose to chemically modulate RLS, and investigated the effect of Sir2p modulating drugs on *C. neoformans* cells in a *Galleria mellonella* infection model. Consistent with our hypothesis that shifts in the generational age of the infecting yeast population alters its vulnerability to host cells, we observed decreased virulence of *C. neoformans* in the *Galleria* host when RLS was prolonged by treatment with Sir2p agonists. In contrast, treatment with a Sir2p antagonist, which shortens RLS enhanced virulence in *Galleria*. In addition, combination of Sir2p agonists with antifungal therapy enhanced the antifungal’s effect. Importantly, no difference in virulence was observed with drug treatment when *sir2Δ* cells were used for infection, which confirmed target specificity and ruled out non-specific effects of the drugs on the *Galleria* host. Thus, this study suggests that RLS modulating drugs, such as Sir2p agonists, shift lifespan and vulnerability of the fungal population, and should be further investigated as a potential class of novel antifungal drug targets that can enhance antifungal efficacy.

## Introduction

*Cryptococcus neoformans* is a formidable fungal pathogen that causes disease in immunocompromised individuals; especially AIDS patients and organ transplant recipients (Perfect and Casadevall, 2002). This haploid fungus grows by asexual reproduction during the course of infection (Alanio et al., 2011). During asexual reproduction, it undergoes asymmetric mitotic divisions, and the sum of these divisions determines its replicative lifespan (RLS) (Steinkraus et al., 2008). In the course of these divisions, the aging mother cells increasingly manifests phenotypic changes, including increased cell body size analogous to changes described in *Saccharomyces cerevisiae, Candida albicans*, and *Schizosaccharomyces pombe* (Fu et al., 2008; Roux et al., 2010; Yang et al., 2011). Also analogous to these yeasts, old *C. neoformans* cells cease to divide at the completion of their RLS (Bouklas et al., 2013). RLS is different from chronological lifespan (CLS) as it involves active growth of the yeast population, whereas CLS is defined as the number of days non-replicating cells remain viable in a medium with no nutrition (Fabrizio and Longo, 2007; Cordero et al., 2011). It is noteworthy that both RLS and CLS affect longevity in *S. cerevisiae*, but their relationship does not always correlate (Qin and Lu, 2006; Fabrizio and Longo, 2007; Barea and Bonatto, 2009).

Recent investigations from our laboratory demonstrated that the RLSs of individual *C. neoformans* strains vary and constitute a stable and reproducible, albeit strain-specific trait (Jain et al., 2009a; Bouklas et al., 2013, 2015). It was also shown that *C. neoformans* undergoes replicative aging during chronic infection in the human host (Alanio et al., 2011). Most importantly, our data from human as well as rat infection indicated that older *C. neoformans* cells accumulate in chronic infection because they were selected. Specifically, old cells were found to be more resistant to hydrogen peroxide stress, macrophage-mediated killing, and amphotericin B-mediated killing (Bouklas et al., 2013). This finding is important because patients with cryptococcosis primarily die from chronic meningoencephalitis (Perfect and Casadevall, 2002), and treatment is commonly initiated after weeks or even months of symptoms. The pathogen’s ability to evade the host immune response combined with its ability to replicate and persist *in vivo* poses a challenge to effective clearance. Consequently, despite the introduction of Combination Antiretroviral Therapy and antifungal therapy, treatment failure, persistent disease, and death remain common (Perfect and Casadevall, 2002; Park et al., 2009).

Based on our published data on selection and acquired resilience of older *C. neoformans* cells (Bouklas et al., 2013), we hypothesized that emergence, selection, and ultimately persistence of older *C. neoformans* cells may constitute an unanticipated virulence trait that could potentially be modulated with drug treatment. Consequently, the intriguing question that has transpired is: could manipulation of RLS in *C. neoformans* have an effect on resilience of the yeast population in the host environment, and therefore indirectly also on virulence? We investigated this question by using drugs known to manipulate RLS. Sirtuins are a large family of NAD^+^-dependent histone deacetylases that are well-conserved across many species (Greiss and Gartner, 2009), including *C. neoformans*. *SIR2* has been implicated in aging in many model organisms (Landry et al., 2000), including *S. cerevisiae* (Kaeberlein et al., 1999).

We demonstrate that chemical agonists and antagonists to Sir2p can result in its activation or inhibition, respectively, and consequently affect the lifespan and resilience of the pathogen population in the host.

## Materials and Methods

### Ethics Statement

All animal experiments were carried out with the approval of the Albert Einstein College of Medicine Institute for Animal Studies. The protocol number 20091015 was approved by the Institutional Animal Care and Use Committee at Einstein. The study was in strict accordance with federal, state, local and institutional guidelines that include “The Guide for the Care and Use of Laboratory Animals,” “The Animal Welfare Act,” and “Public Health Service Policy on Human Care and Use of Laboratory Animals.” All surgery was performed under ketamine and xylazine anesthesia, and every effort was made to minimize suffering.

### Strains and Media

*Cryptococcus neoformans* serotype A VNI strain, H99, was used in this study (J. Perfect, Duke University). Strains stored at -80°C were streaked to single colonies and maintained on Yeast Extract Peptone agar with 0.05%, or 2% dextrose, or 2% dextrose and drug [2.5 mM (Sigma Aldrich) INAM plates and respective NA controls as detailed in a previous publication (McClure et al., 2012), 1 nM resveratrol (Fisher), 1 nM SirAct (BioVision, Inc.), 1 pM SRT1460 (Fisher), 1–10 pM SRT1720 (Fisher), 1 nM Sirtinol (Sigma Aldrich)] before RLS analysis. Standard yeast culture media were employed and described where appropriate. Plasmids pJAF1, pJAF13, and pUC19 were grown in *Escherichia coli* on Luria Bertani (LB) agar plates with ampicillin and have been described elsewhere (Jain et al., 2009b).

### Disruption and Complementation of *SIR2*

The complete ORF sequence of *SIR2* (CNAG_04886.2) obtained from the Broad Institute was replaced with a neomycin cassette in H99 cells by homologous recombination using biolistic transformation in a PDS-1000/He hepta system (Biorad). For transformation, 5 μg of a purified linear DNA construct was used containing neomycin under H99 *ACT1* promoter control and a *TRP1* terminator in addition to 1,000 bp of up- and downstream regions of the target ORF. These regions were amplified from the H99 genomic template using the respective primers (Supplementary Table 2). The neomycin resistance gene was amplified from plasmid pJAF1 using primers Neo-F and Neo-R, and the ampicillin (Amp) resistance gene was amplified from the pUC19 plasmid using primers pUC19-F and pUC19-R. All primers contained a Van91I restriction site to permit one-step directional cloning. Amplified products were restricted with Van91I and ligated using Quick ligase enzyme (New England Biolabs, USA), transformed into XL10 Gold cells (Agilent), and clones were selected on Amp-LB agar plates and confirmed by single digestion with Van91I. Clones with the correct construct were amplified using the primers H99SIR2-Lfor and H99SIR2-Rrev. Transformants were screened on YPD plates containing 100 μg/ml G418 (neomycin) and further confirmed by PCR.

The wt *SIR2* gene was amplified with its native promoter from the H99 genome template with primers H99SIR2R-For and H99SIR2R-Rev containing EcoRV and XhoI restriction sites (Supplementary Table 2). The gene was cloned into plasmid pJAF13, then linearized using ApaI and randomly inserted into *sir2Δ* cells by biolistic transformation. *sir2Δ+SIR2* positive clones were selected on YPD plates containing 100 μg/ml nourseothricin (NAT) (Werner Bioagents, Germany). Gene complementation was confirmed by PCR.

### Lifespan Measurement

Replicative lifespan was measured by microdissection as published in *S. cerevisiae* (Park et al., 2002) with some modifications. Briefly, 20–60 *C. neoformans* cells of each strain were arrayed on an agar plate maintained at 37°C. The first bud of each cell was identified as the virgin mother cell, which then grew in size with every budding event and could be easily distinguished. New buds from the mother cell were separated at the end of each division (1–2 h) using a 50 μm fiber optic needle (Cora Styles) on a tetrad dissection Axioscope A1 microscope (Zeiss) at 100x magnification. The plate was returned to the incubator after each separation, or to 4°C overnight to prevent excessive budding. The study was terminated when cells had failed to divide for 24 h, and then plates were kept incubated for an additional week to ensure that the failure to divide was from death, not cell cycle arrest. The RLS of each cell was the sum of the total buds until cessation of divisions.

Chronological lifespan was determined by adaptation of a *S. cerevisiae* protocol (Burtner et al., 2009). Briefly, 2 × 10^6^ cells/ml of the respective strain were grown in YPD medium for 3 days at 37°C and 150 rpm until they reached stationary phase. They were then transferred to sterile dH_2_O, and the number of viable cells was measured by plating appropriate dilutions every 2 days on YPD agar plates. Colony forming units on the plates were quantitated at 72 h, and the study was terminated when 99.9% of the cells were dead.

### Phenotypic Characterization

For cell and capsule size measurements, *C. neoformans* cells were suspended in India ink. All slides were imaged at 1000X magnification on an Olympus AX70 microscope, pictures were taken with a Qimaging Retiga 1300 digital camera using the Qcapture Suite V2.46 software (Qimaging, Surrey, BC, Canada), and size was measured in Adobe Photoshop CS5 for Macintosh. At least 100 cells were imaged per group. Yeast cells were also stained with mAb 18B7 to the capsular polysaccharide glucuronoxylomannan and visualized with fluorescein isothiocyanate-labeled goat anti-mouse immunoglobulin G (IgG) (Casadevall et al., 1998). Switching frequencies, doubling times, capsule induction, melanization, mating, macrophage-mediated phagocytosis and killing assays, and MICs of amphotericin B were determined as previously described (Jain et al., 2009b).

### Isolation of Old Cells

Wt or mutant *C. neoformans* cells were grown in YPD medium and isolated at 0–2 or 10-generation-old as described previously (Bouklas et al., 2013). Briefly, newly budded *C. neoformans* cells were isolated by elutriation (Beckman JE-5.0 rotor in a Beckman J-6B centrifuge; Beckman Instruments, Inc.) and labeled with Sulfo-NHS-LC-Biotin (Thermo Scientific). The newly budded and labeled cells were grown for several generations (10 generations), and collected by first binding them to streptavidin-conjugated magnetic microbeads (Miltenyi Biotec), then isolating them on a magnetic column (Miltenyi Biotec). Unbound young yeast cells (0-2 generations old) that washed off the column and had been exposed to similar manipulations were used as controls. Purity of old cells was confirmed by fluorescein isothiocyanate (FITC)-staining of the streptavidin-labeled cells.

### Infection Studies

For infection of *Galleria mellonella*, a 10 μl suspension of 2 × 10^3^ or 2 × 10^4^ *C. neoformans* cells was used to infect larvae (*n* = 20–40) (Vanderhorst Wholesale, Inc., St. Marys, OH, USA) in the last proleg as described previously (Cotter et al., 2000). 10 μl of drug [2.5 mM isonicotinamide (Sigma Aldrich), 1 nM resveratrol (Fisher), 1 nM SirAct (BioVision, Inc.), 1 pM SRT1460 (Fisher), 1–10 pM SRT1720 (Fisher), 1 nM Sirtinol (Sigma Aldrich)], or drug and 0.06 μg/ml AMB was given through a different proleg every 2 days.

For infection of mice, 5 × 10^4^ *C. neoformans* cells were used to infect 6–8 week old female BALB/c mice (*n* = 10) (National Cancer Institute, Bethesda, MD, USA) either i.v. or i.t. (Huffnagle et al., 1991; Mukherjee et al., 1994). The fungal burden was determined either 4 h or on day 10 by sacrificing mice, and plating dilutions of homogenized organ suspensions onto YPD plates.

### RNA Sequencing and Analysis

Three biological replicates of wt or *sir2Δ* cells were grown in YPD or YEP and 0.05% glucose broth overnight at 37°C, and approximately 10^8^ cells were collected and suspended in 0.5 mm zirconia beads and RLT buffer (Qiagen), then disrupted mechanically using a mini bead beater (Biospec) for 2 min for a total of four cycles with 1 min intervals on ice. Following lysis, total RNA was isolated using RNeasy mini kit (Qiagen) according to the manufacturer’s instructions. RNA hybridization, data acquisition and analysis were performed by the Genome Technology Access Centre, Washington University in St. Louis (GTAC-WUSTL). Briefly, total RNA was first reverse-transcribed with polyA selection and then sequenced on an Illumina HiSeq 2000. The raw sequence reads were then converted to basecalls, demultiplexed, and aligned to a reference sequence with Tophat v2.0.9 and Bowtie2 v2.1.0. Gene abundances were derived by HTSeq. Differential expression was estimated by pair-wise negative binomial tests with EdgeR and DEXSeq. Gene ontology (GO) enrichment was performed by GTAC-WUSTL. Each gene was assigned a GO category per the Broad Institute’s PFAM annotations using the provided map^1^. Any genes with a *p* < 0.05 by a hypergeometric test and an FDR *q <* 0.25 were used to determine significance. A heatmap of transcriptome data was generated using R software.

### Real-Time PCR

Real-time PCR was performed on RNA isolated from wt or mutant grown in variable media (2% YPD, or 2% YPD with 2.5 mM isonicotinamide, 1 nM resveratrol, 1 nM SirAct, 1 pM SRT1460, 1–10 pM SRT1720, 1 nM Sirtinol). The RNA was cleaned for DNA contamination using the MessageClean kit (GenHunter, Corp.), and cDNA was synthesized using the First-strand Superscript II RT kit (Life Technologies) according to the manufacturer’s instructions. Relative expression of genes was measured by real-time PCR using SYBR green (Applied Biosystems) in an ABI 96 system using primers listed in Supplementary Table 2. Expression levels were performed in quadruplicates and normalized against the wt grown in YPD without drug, and relative transcript levels were determined using the delta-delta CT method. cDNA integrity was verified by measuring expression levels with β-actin, and DNA contamination was ruled out by using cDNA made with dH_2_O.

### Statistics

Standard statistical analysis and non-parametric tests, such as Student’s *t*-test, Log-rank, and Wilcoxon rank sum tests were performed using Prism version 6 (Graphpad), or Microsoft Excel 2011 for Macintosh. Differences were considered significant if *p* < 0.05.

### Data Deposit

The data was deposited at NCBI and can be accessed on GEO (accession #GSE74298).

## Results

### Loss of *SIR2* Shortens the Replicative Lifespan of *C. neoformans*

Sirtuins impact RLS in many diverse organisms, which explains why they are chosen as a target for the development of RLS modulating drugs. Protein sequences encoded by *SIR2* are conserved among fungi (Supplementary Table 1). To test the effect of Sir2p modulating drugs on pathogenesis, a *sir2Δ* strain had to first be generated. *SIR2* was deleted by homologous recombination (Supplementary Figure 1) in a *C. neoformans* serotype A VNI strain, H99, by standard techniques (*sir2Δ*), and a complemented strain was also generated (*sir2Δ+SIR2*). As expected for a lifespan-modulating gene, loss of *SIR2* resulted in measurably impaired fitness. Specifically, a mildly attenuated *in vitro* growth in standard rich media (YPD) was observed (**Table 1**; Supplementary Figure 2A). However, fitness of *sir2Δ* was less affected in calorie restricted (CR) low glucose growth conditions. Of note is that low glucose growth conditions are encountered in the host, especially in the brain environment, and therefore the mutant is still virulent *in vivo*. A smaller capsule size was noted in the mutant; however, both the wild type (wt) and the mutant capsules induced successfully (**Table 1**). Similar to *S. cerevisiae*, the *sir2Δ* mutant was unable to mate with its isogenic mating partner (**Table 1**).

**Table 1:** Characterization of *sir2Δ* phenotypes in *Cryptococcus neoformans* strain H99.

Phenotype	Phenotype in the mutant compared to the wt	Supporting information
Doubling time in YPD	Longer	3.0 vs. 1.9 h (*p* < 0.01)
Doubling time in 0.05% YPD	No measured difference	3.9 vs. 3.2 h
Mating	Sterile	No mating with Kn99*MATa*
Chronological lifespan	Decreased	20 vs. 24 days
Phenotypic switching rate	No switching in either mutant or wt	n/a
Capsule size	Decreased	0.84 + 0.2 vs. 0.96 + 0.2 μm (*p* < 0.01)
Total cell size	No measured difference	6.48 + 0.8 vs. 6.75 + 0.7 μm
Phagocytosis in murine macrophages	No measured difference	81.7 vs. 83.5%
Killing in murine macrophages	No measured difference	76.7 vs. 80.5%
Colony sectoring	No sectoring in either mutant or wt	n/a
GXM staining	Same pattern	Punctate to 18B7 mAb
MIC to amphotericin B	No measured difference	0.25 vs. 0.25 μg/ml
H_2_O_2_ resistance	No measured difference	3.6 vs. 3.5 cm

*Results not significant unless stated otherwise.*

**FIGURE 1:**
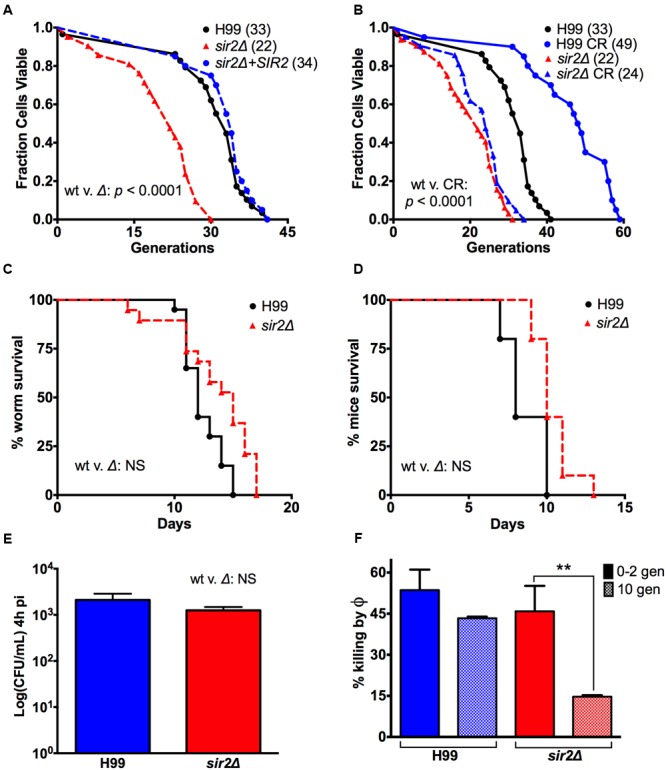
**Loss of *SIR2* shortened the RLS of strain H99, but had no effect on its virulence. (A)** The effect of loss of *SIR2* on the RLS of a serotype A VNI strain, H99, was determined by microdissection of *sir2Δ* cells (dashed line) and found to significantly shorten its median RLS by 33% compared to the wt (straight line). *p* < 0.0001. The shortened RLS was reconstituted in the complement (dotted line). **(B)** 0.05% glucose calorie restriction (CR) significantly extended the median RLS of wt cells by 48%. *p* < 0.0001. CR had no effect on the RLS of *sir2Δ* cells. **(C)** A slightly attenuated virulence was observed in *Galleria mellonella* infected with 2 × 10^3^ mutant cells compared to wt cells. Also **(D)** no significant virulence difference was observed in BALB/c mice injected i.v. with 5 × 10^4^ mutant or wt cells. **(E)** *sir2Δ* cells crossed the blood-brain barrier equally well-compared to the wt strain as suggested by comparable brain CFUs 4 h after i.v. infection. **(F)** 10-generation-old *sir2Δ* cells significantly resisted killing by murine macrophages compared to 0-2 generation old *sir2Δ* cells, and 0–10 generation old wt cells. RLS experiments with the respective medium (*n* = 40–60 cells) were performed at the same time. *p-*values were calculated by Wilcoxon Rank Sum Test. ^∗∗^*p* < 0.01.

Replicative lifespan analysis by microdissection confirmed that *SIR2* controls lifespan in *C. neoformans* (**Figure 1A**). Specifically, lifespan analysis determined that the median RLS of *sir2Δ* cells was shortened by 33% relative to the RLS of H99 cells (33 to 22 generations, *p* < 0.0001). The shortened RLS was reconstituted to 34 generations in the complemented strain. RLS was also determined under CR with 0.05% glucose, whereby 0.05% corresponds to the glucose concentration encountered in human cerebrospinal fluid (CSF). Under CR conditions, the median RLS of H99 cells was extended by 48% from 33 to 49 generations (*p* < 0.0001) (**Figure 1B**). This was dependent on *SIR2*, and accordingly RLS analysis of *sir2Δ* cells under CR demonstrated no effect. CLS, measured by viability without nutrition, was not affected by CR, or different in *sir2Δ* cells compared to the wt (**Table 1**; Supplementary Figure 2B). In summary, similar to *S. cerevisiae, SIR2* function has a major impact on the RLS of *C. neoformans*, but loss also has an effect on its fitness, which has to be taken into consideration when association of lifespan and virulence is examined.

### Loss of *SIR2* Impairs Fitness and Decreases Virulence

Given the impaired fitness, which is seen with most mutants of RLS modulating genes (Kaeberlein and Kennedy, 2005; Kaeberlein et al., 2005), we compared the virulence of *sir2Δ* and wt cells in different infection models, including *G. mellonella* (waxworm) and two murine models of infection. In *Galleria*, survival was found to be slightly decreased at an inoculum dose of 2 × 10^3^ CFU (**Figure 1C**), and significantly decreased at higher inocula (**Figure 3**). Consistent with the observed growth attenuation of *sir2Δ* cells in rich medium, *sir2Δ* cells exhibited hypovirulence also in the murine pulmonary infection model (Supplementary Figure 3), where a lower organ fungal burden (data not shown) was noted in *sir2Δ* infected mice. Interestingly, comparable survival was observed in the intravenous (i.v.) model, where growth difference would be expected to be less pronounced because the brain is a low glucose growth environment (**Figure 1D**). Comparable brain CFUs obtained 4 h after i.v. infection from mouse brain also suggested that both *sir2Δ* and wt cells crossed the blood-brain barrier equally well (**Figure 1E**).

Interestingly, despite the expected hypovirulence, resistance to macrophage-mediated killing at baseline was found to be comparable in the *sir2Δ* and wt cells. This suggests that hypovirulence is predominantly the result of slightly slower growth. And more importantly, when killing was compared in cells aged to 10 generations, resistance was significantly higher in *sir2Δ* cells compared to wt cells of the same generational age (**Figure 1F**). Specifically, 10-generation-old *sir2Δ* cells manifested a higher resistance to killing by a murine macrophage cell line, J774.16, indicating that at 10 generations they were phenotypically older, consistent with their shortened RLS. Whereas, wt cells, which have a longer RLS are still younger phenotypically at 10 generations, which is reflected in their decreased resilience. The reason that this enhanced resilience does not lead to hypervirulence is most likely because young *sir2Δ* cells grow slower than wt cells in the nutrient rich environment of the lung. The *sir2Δ* cells can therefore not expand fast enough to undergo selection for older generations. In summary, these data highlight limitations of knockout mutants and demonstrate that association of RLS and virulence cannot be investigated by a genetic approach because growth, albeit slightly, is impaired by loss of *SIR2*. The data, however, indicate that the mutant is potentially valuable because it can still cause death of *Galleria* and mice under certain experimental conditions. Therefore, it is justified to use the mutant as a control for off-target effects of Sir2p modulating drugs.

### *In vitro* Effects of Sir2p Agonists and Antagonists on RLS

We sought to explore if we could identify drugs that modulate RLS in an individual *C. neoformans* strain. First, we explored RLS modulation *in vitro*. For these experiments, six drugs were chosen. These included isonicotinamide (INAM), a nicotinamide isostere, chosen because it extends RLS in *S. cerevisiae* by alleviating nicotinamide, the feedback inhibitor to Sir2p’s deacetylation function (McClure et al., 2012). Additional Sir2p activators examined were resveratrol, SirAct, and two small molecules (SRT1460 and SRT1720) that were developed originally by Sirtris (now GlaxoSmithKline) to activate the human homolog, Sirt1p. Lastly, we also included a Sir2p inhibitor (sirtinol).

**FIGURE 2:**
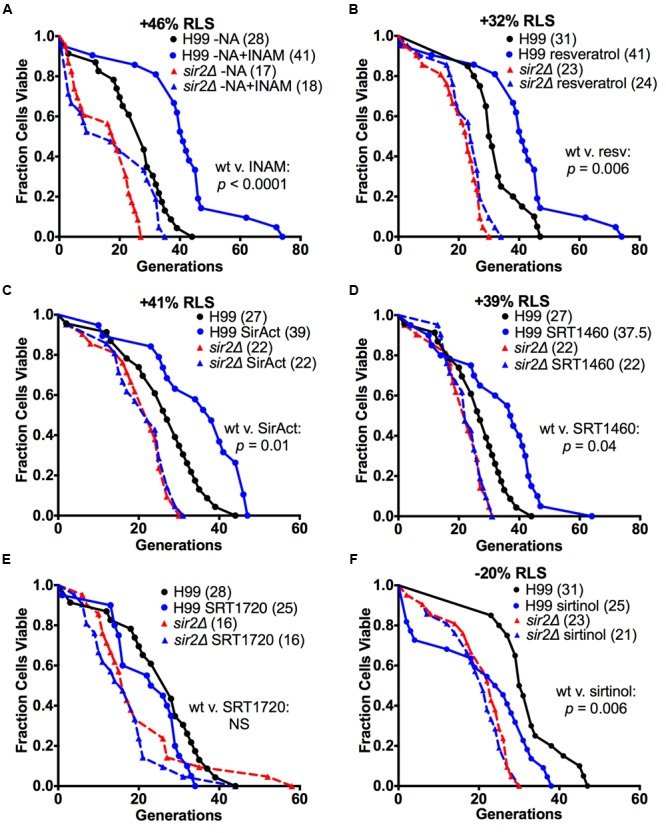
**Multiple Sir2p agonists and an antagonist altered the RLS of H99. (A)** INAM extended the median RLS of H99 cells in NA-depleted conditions by 46%, **(B)** resveratrol by 32%, **(C)** SirAct by 41%, and **(D)** SRT1460 by 39%, respectively (all *p* < 0.04). In contrast **(E)** 1–10 pM SRT1720 failed to extend the median RLS of H99 cells or was toxic to *Cryptococcus neoformans*. As expected, the *SIR2* antagonist **(F)** sirtinol significantly shortened the median RLS of H99 cells by 20% (*p* = 0.006). RLS experiments with the respective drug (*n* = 40–60 cells) were performed at the same time. *p-*values were calculated by Wilcoxon Rank Sum Test.

These studies found that all Sir2p agonists, except SRT1720, had a prolongevity effect on *C. neoformans* (**Figure 2**). Specifically, INAM extended the median RLS of H99 cells restricted for nicotinic acid (NA) by 46% from 28 to 41 generations (*p <* 0.0001) (**Figure 2A**). Resveratrol extended the median RLS of H99 cells by 32% from 31 to 41 generations (*p* = 0.006) (**Figure 2B**). SirAct extended the median RLS of H99 cells by 41% from 27 to 39 generations (*p* = 0.01) (**Figure 2C**). Finally, SRT1460 extended the median RLS of H99 cells by 39% from 27 to 37.5 generations (*p* = 0.04) (**Figure 2D**). SRT1720 was found to be toxic to cells at nM concentrations, and when titered down to non-toxic concentrations of 1–10 pM, it was found to have no significant effect on RLS of H99 cells (**Figure 2E**). As would be expected for Sir2p agonists, RLS prolongation required the presence of *SIR2*, and therefore no prolongevity effect was observed with *sir2Δ* cells for all the RLS drugs tested (**Figure 2**). In addition, we documented the opposite effect on RLS with sirtinol, a Sir2p inhibitor. Exposure to this drug shortened the median RLS of H99 cells by 20% from 31 to 25 generations (*p* = 0.006) in a Sir2p-dependent manner (**Figure 2F**).

### Effects of RLS Modifying Drugs on Virulence in *Galleria*

**FIGURE 3:**
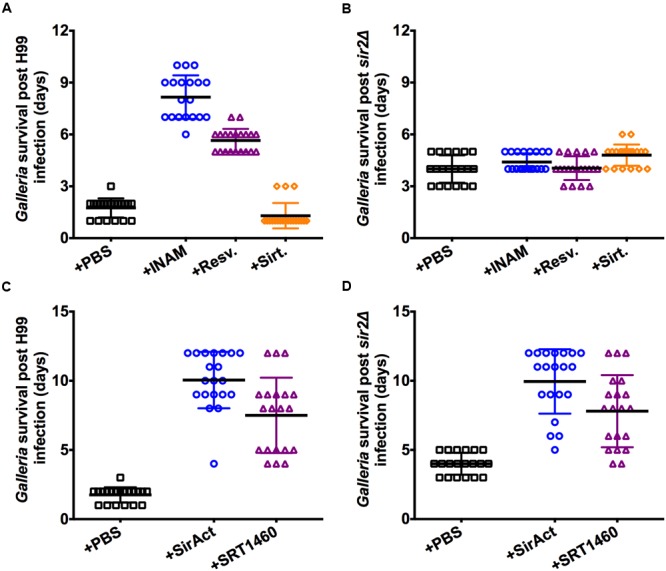
**Sir2p agonists and antagonist alter virulence of H99 in *Galleria*. (A)** INAM and resveratrol given to waxworms infected with 2 × 10^4^ H99 cells resulted in their prolonged survival, whereas sirtinol treatment resulted in decreased survival. **(B)** Lifespan effects were dependent on Sir2p and not observed when infections were done with *sir2Δ* cells. **(C)** SirAct and SRT1460 given to waxworms infected with 2 × 10^4^ H99 cells resulted in their prolonged survival. **(D)** Lifespan effects were dependent on Sir2p and not observed when infections were done with *sir2Δ* cells. Virulence experiments with the respective drug (*n* = 20–40 worms) represent duplicate experiments and were performed with the respective mutant at the same time. *p-*values were calculated by Log-Rank test.

Next the effect of RLS modifying drugs was tested *in vivo*. We chose the *Galleria* infection model because phagocytic cells constitute the predominant host response in this model (Aperis et al., 2007), and drug treatment is easily executed. Older *C. neoformans* cells are not truly more virulent (Bouklas et al., 2013), rather they should be viewed as more resilient to clearance by host cells, and therefore persistence of older cells is the consequence of selective preferential killing of younger cells. We have previously shown that old cells are more resistant to phagocytosis and killing by macrophages (Bouklas et al., 2013). We explored if RLS modulating drugs would affect clearance. Importantly, neither the RLS drugs, nor PBS alone had an effect on non-infected waxworms (Supplementary Figure 4). When INAM was given on alternate days to waxworms infected with H99 at an inocula of 2 × 10^4^ cells, we observed decreased virulence relative to sham-treated waxworms, measured as a significantly prolonged survival of the waxworm (**Figure 3A**). Similar decreased virulence of H99 was observed in waxworms with resveratrol treatment (**Figure 3A**). To control for off target effects, we tested the drugs also in *Galleria* infected with *sir2Δ* cells, where you would not expect an effect if the RLS modulating drug works strictly through Sir2p. As expected because of slightly impaired fitness, the *sir2Δ* infected waxworms die later compared to wt H99 infected waxworms (**Figure 3B**). However, these experiments confirmed specificity because the effect of RLS modulating drugs on waxworm survival was dependent on Sir2p, and neither INAM, nor resveratrol treated waxworms infected with *sir2Δ* cells exhibited a survival difference when compared to untreated controls. Consistent with our hypothesis that prolongation of RLS lessens virulence, whereas shortening of RLS enhances virulence, we found that treatment with the Sir2p inhibitor, sirtinol, had the opposite effect and enhanced virulence, and therefore decreased waxworm survival was documented (**Figure 3A**). This drug effect was again *SIR2* dependent (**Figure 3B**). For waxworms treated with SirAct and SRT1460, significantly increased waxworm survival was also observed (**Figure 3C**), whereas toxicity or no effect was seen with SRT1760 (data not shown). The latter was predicted by the *in vitro* RLS data (**Figure 2**). However, for SRT1460 and SirAct, we found that changes in virulence were not Sir2p-dependent because drug-treated waxworms infected with *sir2Δ* cells also lived significantly longer compared to sham-infected (**Figure 3D**; Supplementary Figure 4).

### Effect on RLS Drugs in Combination with Antifungals

**FIGURE 4:**
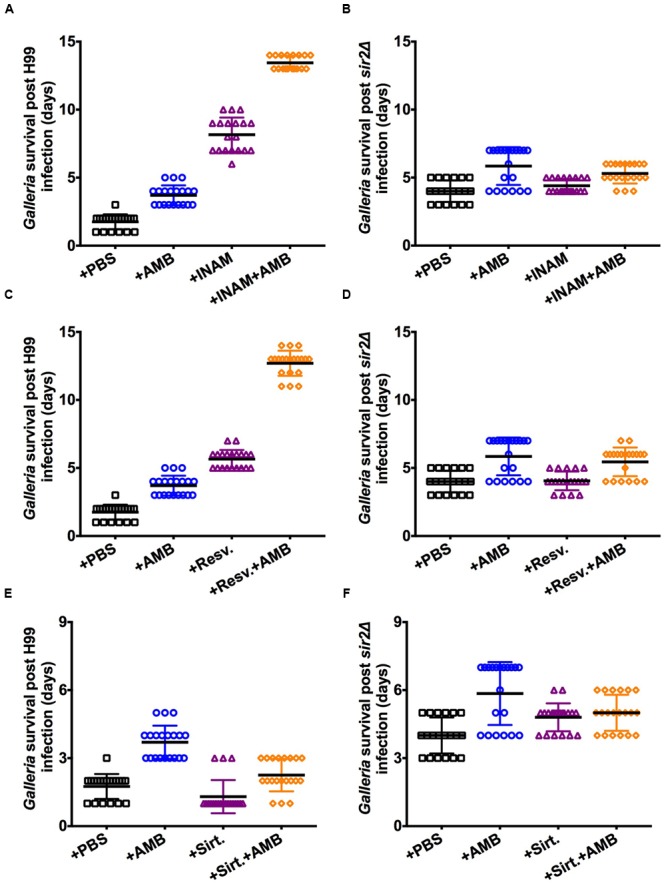
**Adjunctive treatment of Sir2p agonists with amphotericin B enhanced antifungal efficacy.** Treatment with Sir2p agonists, **(A)** INAM or **(C)** resveratrol, and 0.06 μg/ml AMB enhanced antifungal efficacy in H99-infected worms. **(B,D)** Lifespan effects were dependent on Sir2p and not observed when infections were done with *sir2Δ* cells. **(E)** The Sir2p antagonist (sirtinol) had a reverse, albeit minimal, effect that was notably **(F)** Sir2p-dependent. Virulence experiments with the respective drug (*n* = 20–40 worms) represent duplicate experiments and were performed with the respective mutant at the same time. *p-*values were calculated by Log-Rank test.

Next we tested the effect of RLS modulating drugs in combination with antifungal therapy. Previous work demonstrated that sensitivity of *C. neoformans* cells to amphotericin B (AMB) is dependent on the generational age of the cell and enhanced antifungal-efficacy is observed in younger cells (Jain et al., 2009a; Bouklas et al., 2013). Therefore, we tested if concomitant treatment with Sir2p agonists would enhance antifungal efficacy in H99-infected waxworms that were treated with sub-therapeutic levels of AMB. Both INAM (**Figure 4A**) and resveratrol (**Figure 4C**) resulted in significantly increased waxworm survival relative to treatment with RLS or antifungal drug alone. Again, no enhanced survival benefit was observed in the mutant-infected waxworms that received the RLS drug and AMB treatment in combination (**Figures 4B,D**). Here only, AMB treatment prolonged survival. No significant increase in survival was observed with sirtinol-treated waxworms that received AMB (**Figure 4E**); and, this effect was *SIR2*-dependent (**Figure 4F**).

### Effect of RLS Drugs on *SIR2* Expression

**FIGURE 5:**
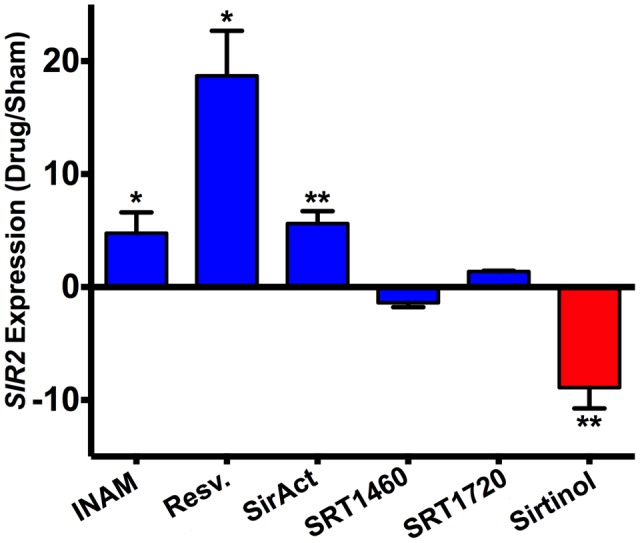
***SIR2* expression was differentially regulated by chemical drugs.** H99 cells grown in the presence of Sir2p agonists (INAM, resveratrol, SirAct) showed a higher expression of *SIR2* as measured by RT-PCR, compared to cells grown in the absence of agonists, or the presence of the Sir2p antagonist, sirtinol. RT-PCR was performed in quadruplicates and normalized to β-actin. *p-*values were calculated by Student’s *t*-test. ^∗^*p* < 0.05, ^∗∗^*p* < 0.01.

Lastly, quantitation of *SIR2* expression by RT-PCR in strains grown with the Sir2p agonists or antagonist confirmed a drug-dependent *SIR2* regulation in H99. It is noteworthy that RLS modulating drugs do not affect doubling times significantly (**Table 2**). Specifically, compared to untreated H99 cells, significant upregulation of *SIR2* expression was documented in response to INAM, resveratrol, and SirAct treatment in H99, and significant downregulation of *SIR2* expression was documented in response to sirtinol (**Figure 5**). *SIR2* expression was not significantly regulated under SRT1460 or SRT1720.

**Table 2:** Doubling times (h) of H99 and *sir2Δ* under RLS-modifying drugs.

Growth conditions	H99	*sir2Δ*
YPD only	3.2	3.8
2.5 mM INAM in YPD	3.1	3.6
1 nM resveratrol in YPD	2.9	3.7
1 nM SirAct	3.4	3.7
10 pM SRT1460	2.8	3.7
10 pM SRT1720	3.3	3.6
1 nM Sirtinol	3.1	3.3

*Results not significant unless stated otherwise.*

### Sir2p is Involved in Several Biological Processes in *C. neoformans* Consistent with the Pleiotropic Effects Observed from *SIR2* Loss

**FIGURE 6:**
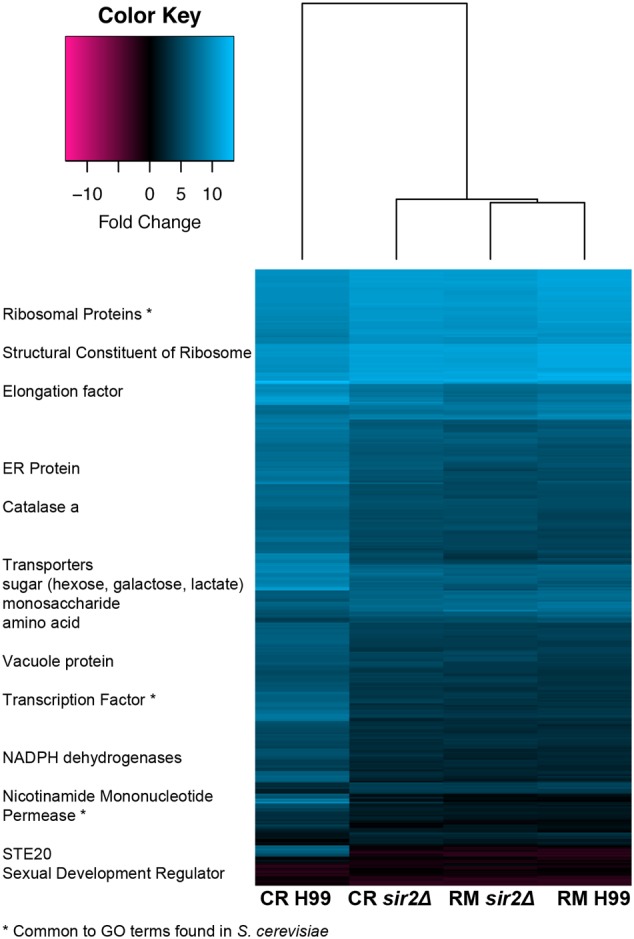
**A loss of *SIR2* in calorie-restricted media affects multiple biological pathways consistent with the observed pleiotropic phenotype.** A heatmap of transcriptomes shows upregulation of ribosome biogenesis genes, transcription and translation, and NAD-regulating genes, as well as downregulation of mating genes. Common gene ontology (GO) categories were also found from similar transcriptome mining in *Saccharomyces cerevisiae* and are highlighted with an asterisk.

Lastly, the transcriptome of *sir2Δ* and wt H99 cells grown in rich media or under more physiologic CR media was compared by RNA sequencing (GEO accession #GSE74298). Grown in rich media (RM: YEP + 2% glucose), 25 genes were down- and 3 genes were upregulated in *sir2Δ* cells (fold change ≤ 1.5-fold, *p* < 0.05). As expected, more pronounced transcriptional regulation was found in CR media (CR: YEP + 0.05% glucose), where 232 genes were down- and 80 genes up-regulated (FC ≤ 1.5-fold, *p* < 0.05). A heatmap was generated (**Figure 6**), and GO analysis was performed to assign the genes affected by *SIR2* loss to GO categories. In rich media, enriched GO categories included the biological processes of vesicle- and ER to Golgi vesicle-mediated transport, intracellular protein transport, and the cellular components of Golgi apparatus, cell division site, and cell tip. In CR growth conditions, GO analysis assigned genes affected by *SIR2* loss to the following distinct biological processes: transmembrane transport, transcription and translation, molecular functions of transporter activity, and the cellular components of cell membrane, ribosome, nucleolus, and mitochondria. Although minimal overlap with respect to specific genes (2%) was noted for the transcriptomes under RM and CR, some common GO pathways overlapped (21%), including transmembrane and transport activity, translation, and the cellular components of mitochondria and ribosomes. Comparison with *S. cerevisiae* transcriptome data (Cherry et al., 2012) identified common GO categories, such as negative regulation of DNA recombination, regulation of DNA-templated transcription, NAD-dependent histone deacetylase activity, and the nucleolus (asterisk in **Figure 6**). Thus, a role of *SIR2* in transport and several intracellular cell components, particularly under nutrient-limiting conditions is suggested and explains the pleiotropic phenotype observed in the mutant.

## Discussion

Recent investigations on replicative aging in *C. neoformans* (Jain et al., 2009a; Bouklas et al., 2013) indicate that older *C. neoformans* cells of advanced replicative age are selected *in vivo* during chronic infection. Furthermore, data indicated that old cells are selected because their phenotype is more resilient in the setting of chronic disease. Accordingly, we demonstrated that 10-generation-old cells were more resistant to antifungal therapy and phagocytic killing. In this study, we present evidence that modulation of RLS in the *C. neoformans* strain, H99, through treatment with Sir2p agonists changes the vulnerability/resilience of the pathogen population, and therefore an impact on virulence, as well as sensitivity to antifungal therapy is observed.

The question of whether a shortened or extended RLS confers a benefit to a *C. neoformans* strain is challenging to investigate because lifespan is a dynamic trait that has to first emerge, and in addition lifespan (Bitterman et al., 2003; Jazwinski, 2004; Steinkraus et al., 2008; Bouklas et al., 2013, 2015), and virulence (Adler et al., 2011; Haynes et al., 2011; Kronstad et al., 2012; Zaragoza and Nielsen, 2013; Sabiiti et al., 2014) are regulated by multiple factors. A straightforward reductionist approach, where RLS is modified through loss of a longevity-promoting gene, is therefore not feasible. In addition, a genetic approach is hampered by the fact that longevity-regulating genes in *S. cerevisiae* also regulate fitness (Kaeberlein and Kennedy, 2005; Kaeberlein et al., 2005). This is also true for known homologs in *C. neoformans*; for instance, the *tor1Δ* mutant is not viable (Cruz et al., 2001), the *ras1Δ* mutant grows slower (Waugh et al., 2002), and the *sch9Δ* mutant has an altered polysaccharide capsule (Wang et al., 2004), and would not constitute as adequate targets to answer that question. Therefore, changes in virulence in these *C. neoformans* mutants cannot be related to changes in RLS.

*SIR2* homologs are among the most intensely investigated lifespan modulating genes. In *S. cerevisiae*, loss of *SIR2* shortens RLS, while overexpression results in extension of RLS (Kaeberlein et al., 1999). *SIR2* prolongevity effects have been reported in other eukaryotes (Tissenbaum and Guarente, 2001; Wood et al., 2004; Guarente, 2007), and drugs that alter Sir2p function are being actively pursued (Baur et al., 2012). However, Sir2p is a histone deacetylase (Guarente and Kenyon, 2000) that regulates over 100 genes in *S. cerevisiae* (Cherry et al., 2012). Pleiotropic effects in the *sir2Δ* also include loss of fitness (Kaeberlein and Kennedy, 2005; Kaeberlein et al., 2005).

In this study, we first investigated the loss of Sir2p function in the standard serotype A VNI strain, H99, which is derived from a patient and used for experiments by the majority of laboratories that investigate *C. neoformans*. Consistent with RLS studies in *S. cerevisiae* (Lin et al., 2000; Kaeberlein et al., 2004), *sir2Δ C. neoformans* cells exhibited a significantly shortened median RLS that was regained with reconstitution. As predicted by *S. cerevisiae* and other model organisms of aging (Wood et al., 2004; Guarente and Picard, 2005; Fontana et al., 2010; Skinner and Lin, 2010), CR was found to prolong lifespan in *C. neoformans* as well. CR is modeled in yeast by reduction of glucose content from 2 to 0.05% (Lin et al., 2002; Kennedy et al., 2005). Extension of lifespan through CR was dependent on *SIR2* in H99. Accordingly, *SIR2* was upregulated in H99 cells under CR. It is noteworthy that so far, the majority of lifespan studies under CR have been conducted in primarily fermentative Crabtree-positive yeasts, such as *S. cerevisiae* and *Schizosaccharomyces pombe*, and only a few studies in the primarily respiratory Crabtree-negative yeasts, *Candida albicans* and *Kluyveromyces lactis*, are emerging (Skinner and Lin, 2010). *C. neoformans* is an obligate aerobic yeast, but can tolerate some hypoxic stress (Chun et al., 2007). CR in Crabtree-negative and in obligate aerobic yeasts does not activate a fermentation-to-respiration switch. Thus, *C. neoformans* provides a unique platform to study the respiration switch-independent mechanisms of CR.

Sirtuins have been implicated in a wide range of cellular processes beyond aging. Our transcriptome data confirm this. We found that genes involved in many diverse biological processes are regulated. As expected, regulation is greatly enhanced under CR conditions. Important virulence-associated traits that were altered in H99 *sir2Δ* cells include a mating defect and impaired growth, which is physiologically more relevant in the host environment. Notably the growth defect was not significant under CR growth conditions. Other virulence-associated properties, such as melanization, H_2_O_2_ resistance, phagocytosis, and killing in macrophages were not affected by loss of *SIR2* in young cells. The capsule difference was judged as minor, and capsule was inducible regardless of *SIR2* loss. Impaired growth in the mutant cells underscores the aforementioned predicament, namely that RLS mutants cannot be used to determine if the length of RLS affects virulence. Fortunately, the *sir2Δ* was virulent in the *G. mellonella* infection model despite mildly attenuated growth, which allows us to use this mutant as a valuable control. Even in rodents, the *sir2Δ* mutant could be used as a control in the CNS model because it is virulent and can cause death in mice.

**FIGURE 7:**
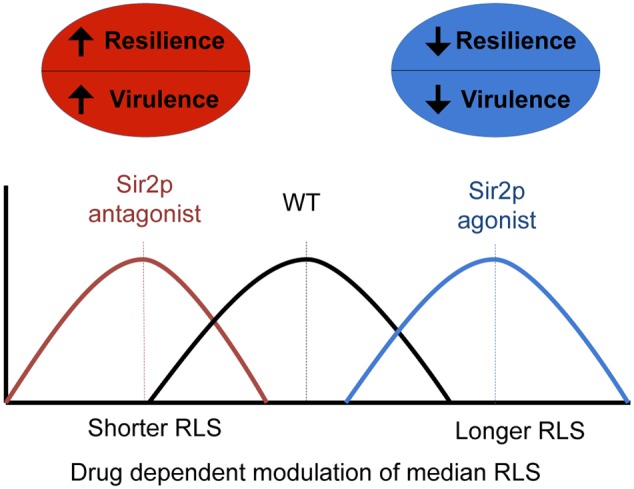
**A schemata highlights the relationship between virulence and resilience of the pathogen population, and the effect of RLS modulating drugs on lifespan**.

Over the past two decades, genetic approaches using diverse organisms have identified 100s of aging genes and highlighted evolutionary conservation among longevity pathways between disparate species (Managbanag et al., 2008). Although the major driving force of aging research is its application to novel therapies against chronic disease and direct extension of human lifespan, our intention was to test *SIR2* modifying anti-aging drugs with respect to their ability to alter the median RLS of eukaryotic pathogen populations. Given that cells of advanced generational age exhibit enhanced resistance to phagocytic killing, and to antifungals (Jain et al., 2009a; Bouklas et al., 2013), it was reasonable to hypothesize that in a *C. neoformans* strain with a prolonged RLS, the resilient old age phenotype would emerge later, and therefore would contribute to decreased resilience and virulence of the pathogen population (**Figure 7**).

Significantly, increased longevity was achieved in H99 cells *in vitro* with four of the five tested Sir2p agonists. As expected, the opposite effect on RLS was observed with the Sir2p inhibitor, sirtinol. SRT1460 and SRT1720 are high affinity small molecules that were designed to bind to the human Sir2p analog, Sirt1p (Milne et al., 2007), and therefore it was not surprising that SRT1720 did not prolong RLS in *C. neoformans*. SRT1460 had a statistically significant prolongevity effect. Both did not induce fungal *SIR2 in vitro*. Resveratrol, a stilbenoid, is an established anti-aging drug that has a significant prolongevity effect on the RLS of *S. cerevisiae* (Howitz et al., 2003), and also on other model organisms (Baur et al., 2006; Bass et al., 2007). In *C. neoformans*, we documented a significant prolongevity effect on RLS as well. The prolongevity effect of resveratrol in *S. cerevisiae* is *SIR2*-independent (Kaeberlein and Kennedy, 2007; McClure et al., 2012), but this is strain dependent. Our data, however, indicate that for the *C. neoformans* strain, H99, the prolongevity effect was dependent on *SIR2.* Future studies with the *sir2Δ* in other *C. neoformans* strain backgrounds would have to be done to confirm consistent dependence on *SIR2*. INAM is a nicotinamide isostere that extends RLS in *S. cerevisiae* by alleviating nicotinamide, an NAD+ precursor and feedback inhibitor to Sir2p’s deacetylation function (McClure et al., 2012). This drug is thought to extend RLS only through the action of Sir2p in *S. cerevisiae*. In *C. neoformans*, this was confirmed. SirAct is a carboxamide, which was developed to treat aging related diseases in humans (Nayagam et al., 2006). Our results demonstrate that this drug also has a significant Sir2p dependent effect on *C. neoformans* RLS.

Based on our *in vitro* data, we sought to test the effect of RLS modulating drugs in an *in vivo* virulence model in *Galleria*. Indeed, these data confirmed that RLS modulating drugs could have an impact on virulence. We demonstrated that RLS prolonging drugs increase survival and decrease virulence in *Galleria*, whereas RLS shortening drugs decrease waxworm survival when infected with H99 wt cells. In addition, our experiments showed that RLS modulating drugs could enhance the antifungal efficacy of amphotericin B in *C. neoformans* infected *Galleria*. This effect is not seen when sirtinol, which shortens RLS, or in all the cases where waxworms were infected with *sir2Δ* cells instead of the wt. We propose that prolongation of RLS alters vulnerability *in vivo* as it shifts the median RLS to a younger pathogen population, which has not acquired the old age phenotype yet.

One concern is that the drugs could have an independent effect on the host’s virulence. Resveratrol, for instance, inhibits laccase activity and melanization in *C. neoformans* cells (Fowler et al., 2011). However, the fact that *sir2Δ* infected *Galleria* do not exhibit the same changes in virulence suggests that the decreased virulence is dependent on fungal-specific Sir2p. It is also noteworthy that doubling times are not significantly affected by drug treatment. RNA transcriptome comparison of H99 in CR conditions demonstrates upregulation of *SIR2* under CR. Most importantly, published transcriptome data from CNS derived *C. neoformans* cells (Chen et al., 2014) also demonstrate that *SIR2* is upregulated in yeast derived from the CSF. Hence, future studies in rodent models constitute a rational approach to further explore this expanding class of drugs (Zhai et al., 2012). An additional concern is that the drugs could have an independent effect on mammalian cells, which share the homologous Sirt1p. Recent successful designs of human specific Sirt1p agonists suggest that with proper medicinal chemistry (Nayagam et al., 2006; Milne et al., 2007), it may be possible to produce fungal-specific Sir2p analog(s) that have minimal off-target effects. Our data with *Galleria* suggest that SRT1460 has off-target and Sir2p-independent effects on the host that may affect survival. This effect, however, is not observed for INAM or resveratrol.

Finally, our data further support a more complex understanding of pathogenesis, whereby the median RLS of a strain may not matter *per se*, but age-related resilience should be viewed as an emerging virulence trait of a pathogen population that may come as a trade-off for fitness. This naturally acquired old age phenotype, once selected could become dominant in the pathogen population, and indeed impact outcome and affect persistence (**Figure 7**). Our data strongly suggest that this process can be harnessed and targeted with drugs, which opens up a new class of antifungal drug targets. Importantly, this novel concept of generational phenotypes that promotes their selection within a pathogen population may be relevant to other eukaryotic pathogen populations, many of which cause chronic diseases that are notoriously difficult to treat, and for which new drug targets are desperately required. Aging related phenotypes are not present in overnight cultures and only become relevant in the host environment because a highly selective host response has to be present *in vivo* to drive selection, and permit the emergence of older cells in the host.

## Author Contributions

BF, TB, and NJ conceived and designed the work. TB performed the experiments and collected the data. BF, TB, and NJ performed data analysis and interpretation. BF and TB drafted and revised the article. BF, TB, and NJ gave final approval of the version to be published.

## Conflict of Interest Statement

The authors declare that the research was conducted in the absence of any commercial or financial relationships that could be construed as a potential conflict of interest.

## Footnotes

1 http://www.geneontology.org/external2go/pfam2go
