# Supplementary material for: Modulation of Replicative Lifespan in Cryptococcus neoformans: Implications for Virulence
Source: Front Microbiol. 2017 Jan 30;8:98. doi: 10.3389/fmicb.2017.00098 (PMC5276861; doi:10.3389/fmicb.2017.00098)
Supplement: Supplementary file 1 [file Data_Sheet_1.DOCX]

***Supplementary Material***

**Tejas Bouklas^1,2^, Neena Jain^4^, and Bettina C. Fries^2,3,4,5^***

***Correspondence:** Bettina C. Fries bettina.fries@stonybrookmedicine.edu

Supplementary Figures and Tables


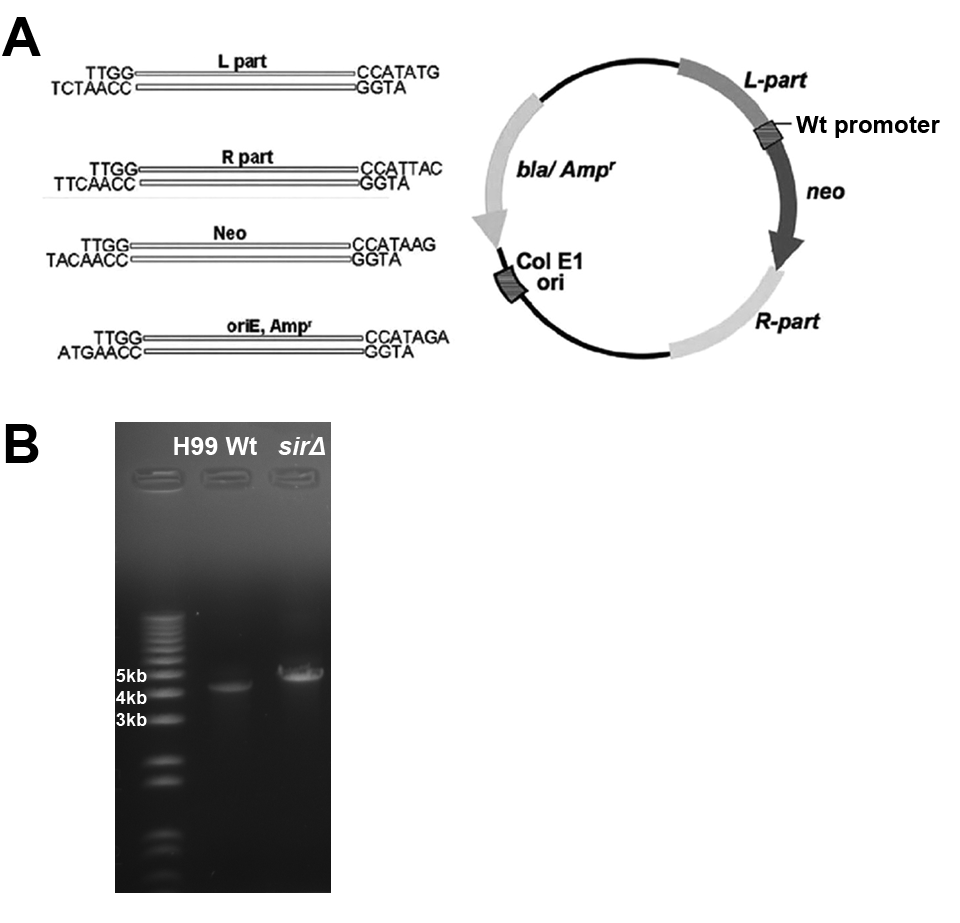


**Supplementary Figure 1. Confirmation of the *sir2Δ* mutant.** (**A**) Plasmid construct used to generate homologous recombinants of *sir2Δ* cells in the H99 background. Van91I restriction sites on the individual primers were used for rapid directional cloning. (**B**) PCR amplification showed that there was correct homologous recombination in the mutant (*sir2Δ*) compared to the wt strain (H99).


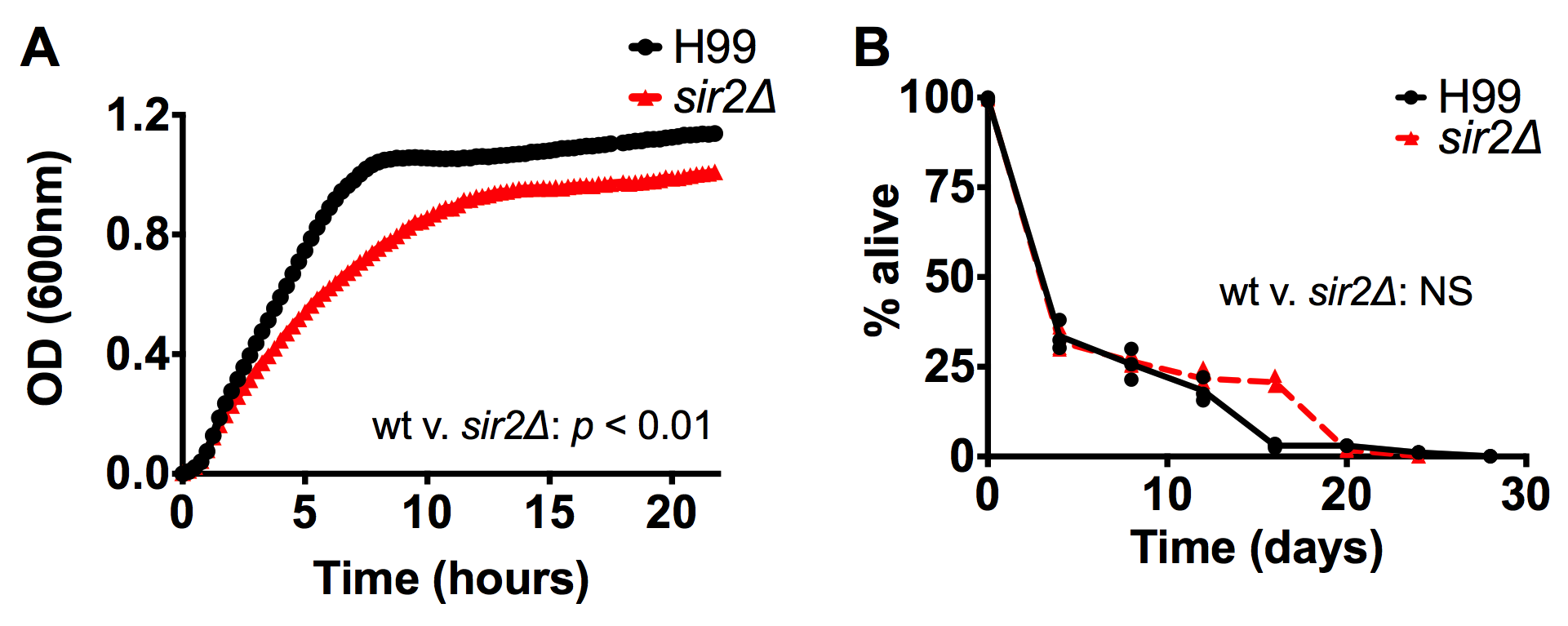


**Supplementary Figure 2. Phenotypic characterization of *sir2Δ*** **and wt.** (**A**) Doubling time measured by growth curves of H99 wt or *sir2Δ* cells in YPD. Experiments were done in triplicates and significance was calculated by Student’s t-test. (**B**) Chronological age measured by time viable in water for H99 wt or *sir2Δ* cells in rich media. Experiments were done in triplicates, and significance was calculated by Student’s t-test.


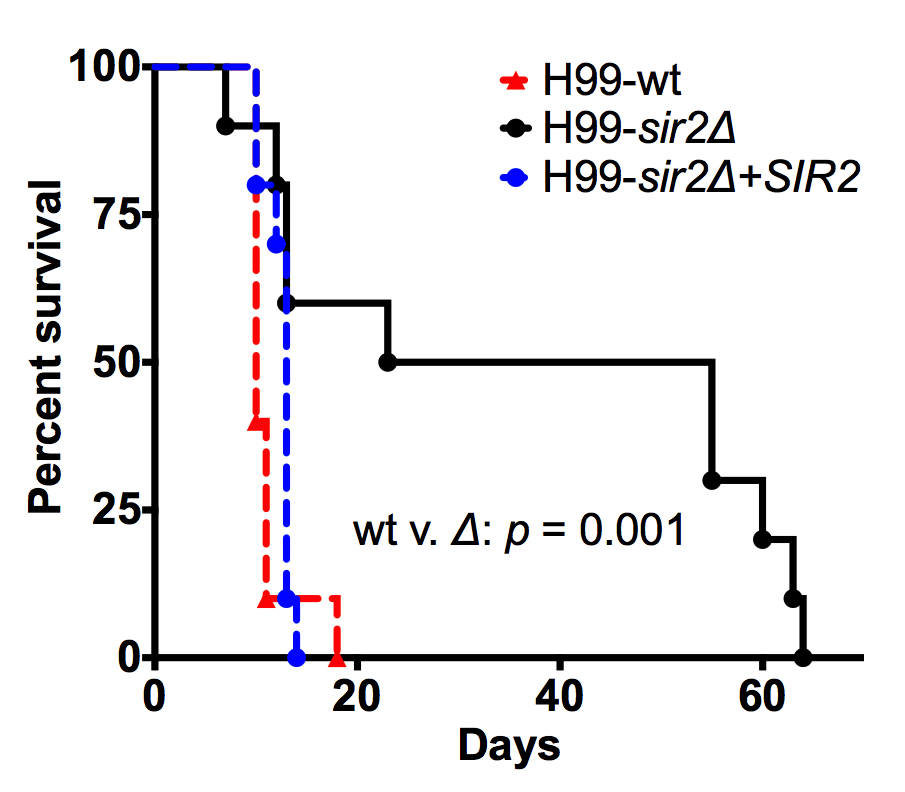


**Supplementary Figure 3. Loss of *SIR2* resulted in hypovirulent *C. neoformans* in the pulmonary murine infection model.** BALB/c mice (n=10) infected intratracheally (i.t.) with *sir2Δ* survived longer than those infected with the respective wt strain (*p =* 0.001 by Log-Rank Test). The loss in virulence was rescued by the complemented strain.


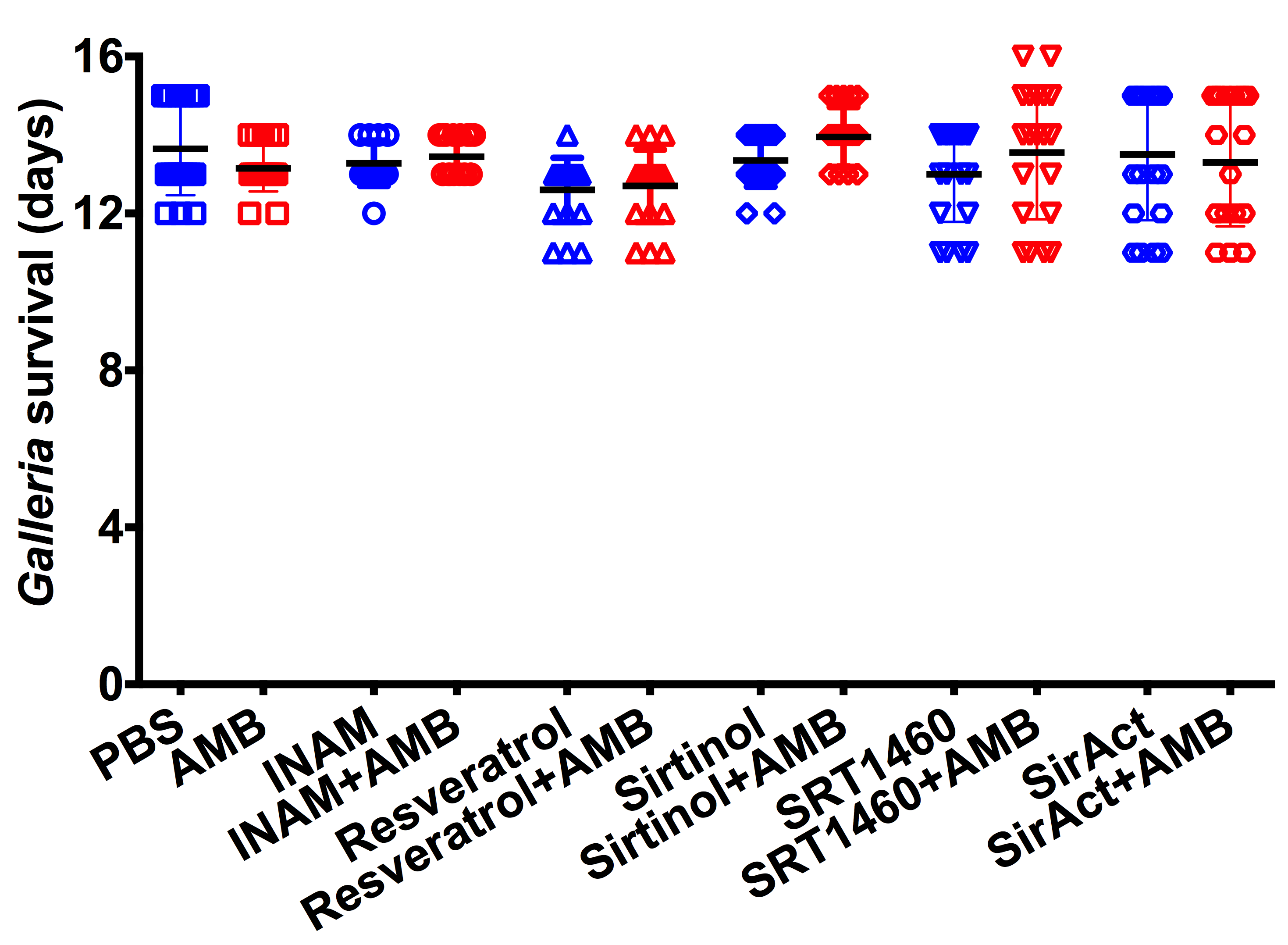


**Supplementary Figure 4. *Galleria* survival was not affected by drugs alone.** *Galleria* survival was not significantly affected when injected with the drugs (INAM, resveratrol, sirtinol, SRT1460, SirAct) either alone, or in combination with sub-therapeutic AMB. Also, PBS or sub-therapeutic AMB alone had no significant effect on *Galleria* survival. Experiments were done in duplicates (n = 20-40 worms), and significance was calculated by Log-Rank test.

**Supplementary Table 1. *SIR2* is conserved in fungi**

|  | **Size** | **Chromosome** | **Protein Homology  to H99** |
| --- | --- | --- | --- |
| *Cryptococcus neoformans* (H99) | 596 aa | 10 | - |
| *Cryptococcus neoformans* (RC2) | 596 aa | 10 | 99% |
| *Schizosaccharomyces pombe* | 475 aa | 2 | 41% |
| *Saccharomyces cerevisiae* | 562 aa | 4 | 53% |
| *Candida albicans* | 519 aa | 2 | 49% |
| *Candida glabrata* | 519 aa | K | 43% |

**Supplementary Table 2. List of primers used in the study.**

| **Name** | **Purpose** | | **Sequence (5’- 3’)** |
| --- | --- | --- | --- |
| Neo-F  Neo-R | Amplification of neomycin cassette | CCATATGTTGGTAA AACGACGGCCAGTGAATTGTA  CCATGAATTGGCAGGAAACAGCTATGACCATGATT | |
| pUC19-F  pUC19-R | Amplification of origin of replication and ampicillin resistance gene | CCATTTTTTGGGAAAGGGCCTCGTGATACGCCT  CCATTCTTTGGGCTTTCCAGTCGGGAAACCTGT | |
| H99SIR2-Lfor  H99SIR2-Lrev | Amplification of 1000 nucleotides upstream of *SIR2* in H99-wt | CCATAGATTGGCCCAGCCCCAATTATCTTCT  CCATCATTTGGAGACGCCATTTCCACCTTCT | |
| H99SIR2-Rfor  H99SIR2-Rrev | Amplification of 1000 nucleotides downstream of *SIR2* in H99-wt | CCATTTCTTGGCGACAGCGACCTACAGGAAC  CCATAAATTGGGCCAGAGTAATGTATCGTGCTG | |
| H99SIR2R-For  H99SIR2R-Rev | Complementation of *SIR2* in H99-wt | CTCGAGCCCAGCCCCAATTATCTTCT TCTAGAGCCAGAGTAATGTATCGTGCTG | |
| SIR2For  SIR2Rev | Measurement of mRNA expression of *SIR2* | CCAAGTCTAAGAAGATCATT  CTTGGCGAAGGAGTAGAAAA | |
